# Author Correction: Germline mutations in mitochondrial complex I reveal genetic and targetable vulnerability in IDH1-mutant acute myeloid leukaemia

**DOI:** 10.1038/s41467-022-31952-7

**Published:** 2022-07-15

**Authors:** Mahmoud A. Bassal, Saumya E. Samaraweera, Kelly Lim, Brooks A. Benard, Sheree Bailey, Satinder Kaur, Paul Leo, John Toubia, Chloe Thompson-Peach, Tran Nguyen, Kyaw Ze Ya Maung, Debora A. Casolari, Diana G. Iarossi, Ilaria S. Pagani, Jason Powell, Stuart Pitson, Siria Natera, Ute Roessner, Ian D. Lewis, Anna L. Brown, Daniel G. Tenen, Nirmal Robinson, David M. Ross, Ravindra Majeti, Thomas J. Gonda, Daniel Thomas, Richard J. D’Andrea

**Affiliations:** 1 Harvard Stem Cell Institute, Harvard Medical School, Boston, USA; 2 Cancer Science Institute of Singapore, National University of Singapore, Singapore, Singapore; 3 Centre for Cancer Biology, University of South Australia and SA Pathology, Adelaide, Australia; 4 Discipline of Medicine, University of Adelaide, Adelaide, Australia; 5 Hematology Division, Department of Medicine, Stanford Cancer Institute, Institute for Stem Cell and Regenerative Medicine, Stanford University, Stanford, USA; 6 Clinical and Health Sciences, University of South Australia, Adelaide, Australia; 7 Diamantina Institute, Translational Research Institute, Brisbane, Australia; 8 Precision Medicine Theme, South Australian Health and Medical Research Institute, Adelaide, Australia; 9 Metabolomics Australia, The University of Melbourne, Melbourne, Australia; 10 Adelaide Oncology & Haematology, Adelaide, Australia; 11 Department of Genetics and Molecular Pathology, SA Pathology, Adelaide, SA Australia; 12 Department of Clinical Haematology, Royal Adelaide Hospital, Adelaide, Australia; 13 School of Pharmacy, University of Queensland, Brisbane, Australia

**Keywords:** Cancer genetics, Cancer metabolism, Acute myeloid leukaemia

Correction to: *Nature Communications* 10.1038/s41467-022-30223-9, published online 12 May 2022.

In this article the author name Brooks A. Benard was incorrectly written as Brooks A. Bernard. The original article has been corrected.

