# Supplementary material for: Germline mutations in mitochondrial complex I reveal genetic and targetable vulnerability in IDH1-mutant acute myeloid leukaemia
Source: Nat Commun. 2022 May 12;13:2614. doi: 10.1038/s41467-022-30223-9 (PMC9098909; doi:10.1038/s41467-022-30223-9)
Supplement: Supplementary file 1 — Supplementary Information [file 41467_2022_30223_MOESM1_ESM.pdf]

**Supplementary Information**

# Germline mutations in mitochondrial complex I reveal genetic and targetable vulnerability in IDH1-mutant Acute Myeloid Leukemia

Mahmoud A. Bassal[1,2*], Saumya E. Samaraweera[3*], Kelly Lim[4], Brooks A Bernard[5], Sheree Bailey[6], Satinder Kaur[4], Paul Leo[7], John Toubia[3], Chloe Thompson-Peach[4], Tran Nguyen[3], Kyaw Ze Ya Maung[3], Debora A. Casolari[3], Diana G. Iarossi[3], Ilaria S. Pagani[8], Jason Powell[3], Stuart Pitson[3], Siria Natera[9], Ute Roessner[9], Ian D Lewis[10], Anna L Brown[3,6,11], Daniel G. Tenen[2,1], Nirmal Robinson[3], David M. Ross[3,4,8,12], Ravindra Majeti,[5] Thomas J. Gonda,[6,13] Daniel Thomas[4,5,8] and Richard J D'Andrea[3].

1. Harvard Stem Cell Institute, Harvard Medical School, Boston, USA
2. Cancer Science Institute of Singapore, National University of Singapore, Singapore
3. Centre for Cancer Biology, University of South Australia and SA Pathology, Adelaide, Australia
4. Discipline of Medicine, University of Adelaide, Adelaide, Australia
5. Hematology Division, Department of Medicine, Stanford Cancer Institute, Institute for Stem Cell and Regenerative Medicine, Stanford University, USA
6. Clinical and Health Sciences, University of South Australia, Adelaide Australia
7. Diamantina Institute, Translational Research Institute, Brisbane, Australia.
8. Precision Medicine Theme, South Australian Health and Medical Research Institute, Adelaide, Australia
9. Metabolomics Australia, The University of Melbourne, Melbourne, Australia
10. Adelaide Oncology & Haematology, Adelaide, Australia
11. Department of Genetics and Molecular Pathology, SA Pathology, Adelaide, South Australia, Australia
12. Department of Clinical Haematology, Royal Adelaide Hospital, Adelaide, Australia
13. School of Pharmacy, University of Queensland, Brisbane, Australia

*These authors contributed equally to this work.

Correspondence: richard.dandrea@unisa.edu.au

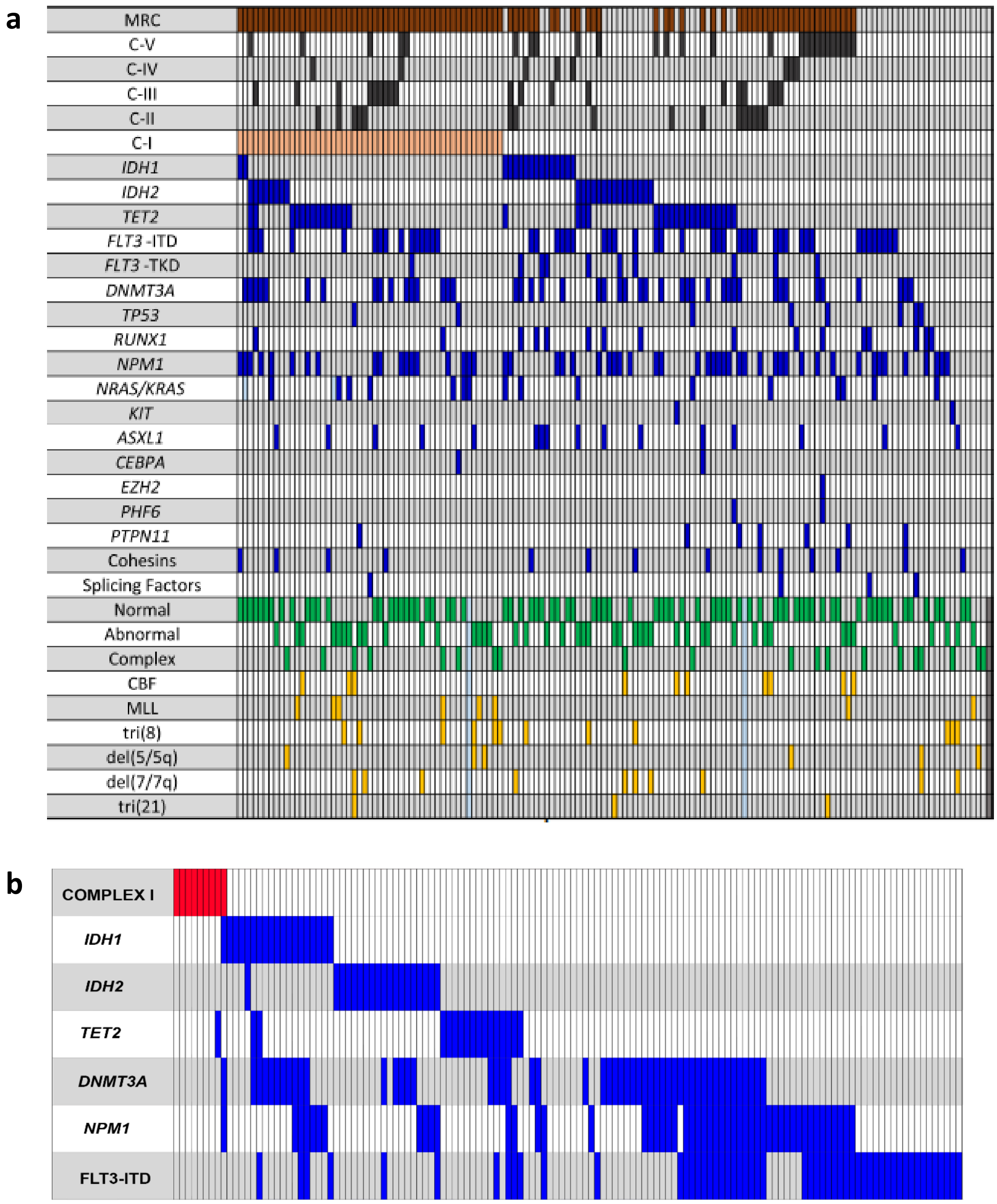


**Supplementary Figure 1. Interaction between complex I variants and *IDH1* mutations. (a)** Rare mitochondrial respiratory chain (MRC) variants and common AML mutations in the Australian whole exome sequencing AML cohort (n=145). Each column represents an AML diagnostic sample. Shaded cells represent an identified rare MRC variant or a mutation in the gene shown. Light blue shading represents unknown status. Cohesins and splicing factors are as determined in The Cancer Genome Atlas (TCGA)[1]. Complex karyotype is classified as 3 or more abnormalities. CBF includes inv(16), del(16), t(16;16) and t(8;21). MLL includes translocations involving 11q23. **(b)** Mutation profile showing somatic mitochondrially-encoded complex I variants for the TCGA AML cohort[1]. The mitochondrial-encoded complex I variants show segregation from *IDH1* R132 mutations (FDR 0.03, weighted exclusivity test).

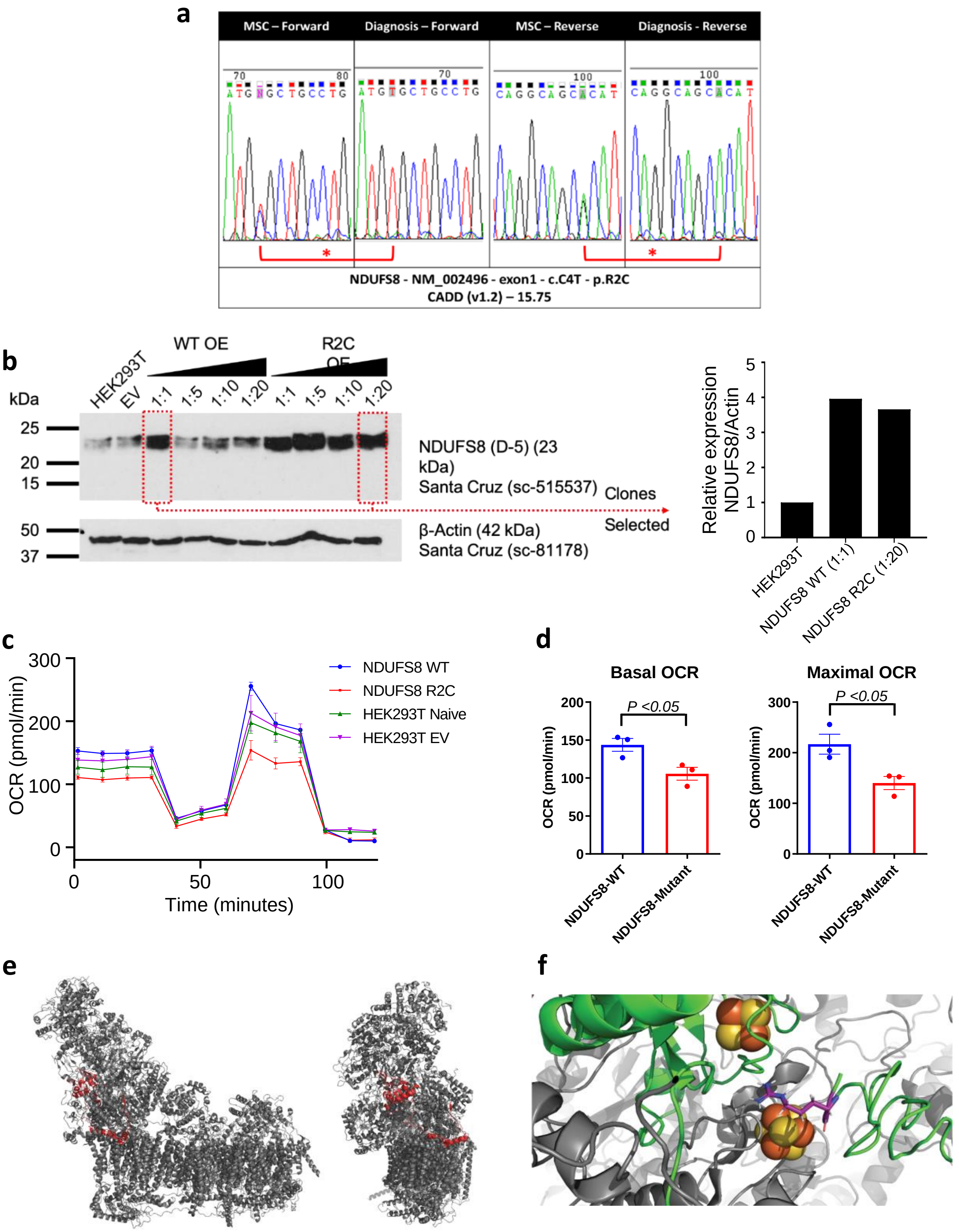


**Supplementary Figure 2. Characterisation of NDUFS8 p.R2C variant. (a)** PCR amplification of the genomic locus and Sanger sequencing of both mesenchymal stromal cells (MSC, germline) and diagnostic bone marrow samples revealed a heterozygous germline variant (detected in both forward and reverse directions), with a loss-of-heterozygosity of the wild-type allele in the diagnostic sample. Sequencing primers that were used are forward: TCAGTGGAGACTTGGGATCC and reverse: AAAAGAAGGGCGTGACGTC. **(b)** Western

blot showing NDUFS8 protein abundance in HEK293T cells over-expressing wild type (WT) NDUFS8 or the R2C mutant compared to cells with empty vector (EV), and naïve HEK293T. Representative blot of two independent experiments is shown. Bar plot shows quantified abundance of NDUFS8 protein in selected clones relative to β-Actin. Abundance quantified using Image Lab version 6 (Bio-Rad Laboratories). The uncropped scan of the blot and quantitation are provided in the Source Data file. **(c)** Oxygen consumption rate (OCR) in HEK293T cells expressing exogenous NDUFS8 R2C mutant vs WT showing decreased basal and maximal OCR for clones selected in b. Each point represents the mean of a given measurement timepoint and error bars show +/- SD of triplicate readings from a single representative experiment (of n=3). **(d)** Basal and maximal OCR for HEK293T cells expressing NDUFS8 R2C mutation vs WT. Data are presented as mean +/- SEM of 3 independent experiments. Each dot represents an independent experiment. Statistical significance was determined by unpaired two-tailed t-test. Significance values for basal: $P = 0.034$ and maximal: $P = 0.0317$. Source data are provided in Source Data file. **(e)** Using the crystal structure of complex-I from *Bos taurus* (Protein Data Bank ID 5XTH), the spatial location of NDUFS8 (red) was determined within the matrix arm of complex I. **(f)** Location of the wild-type Arginine[2] with respect to the iron-sulphur cluster catalytic cores of complex I. Utilizing the predicted structure of NDUFS8 (green), the location of the mutated arginine residue (p.R2) was identified (purple). Spatial localization places this arginine residue in contact with an Fe-S catalytic core within complex I (yellow and orange structure). Mutation of the arginine to a cysteine (p.R2C), as identified in AML, is predicted to lead to decreased stability of the redox active Fe-S cluster and less efficient electron transfer.

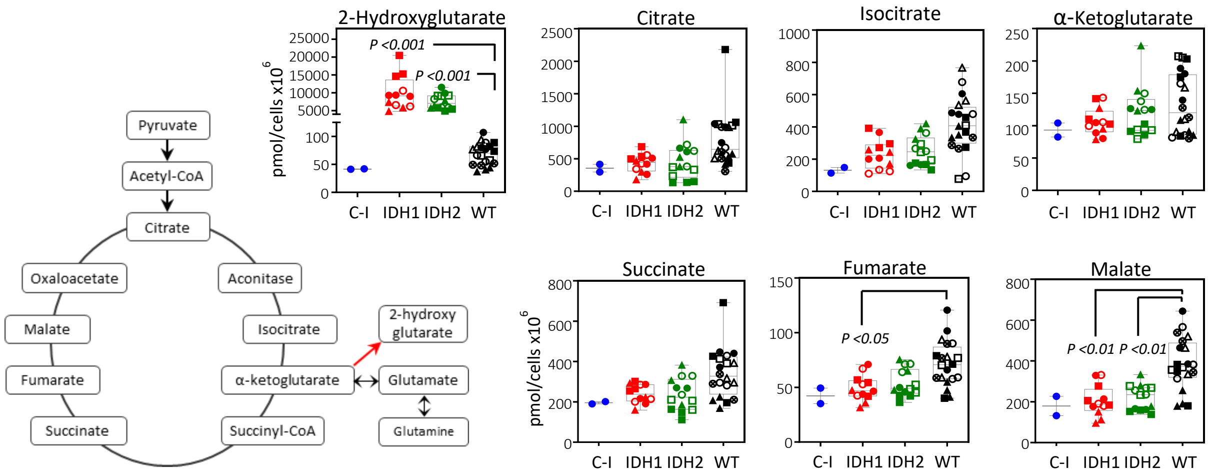


**Supplementary Figure 3. Characterisation of Krebs cycle metabolites in primary AML samples.** Metabolite concentrations determined for an AML sample with the complex I (C-I) variant NDUFS8 p.R2C, and for *IDH1*- (n=4) and *IDH2*-mutant (n=5) samples, and samples wild type (WT) for *IDH1* and *IDH2* (n=7). Metabolite concentrations were determined in duplicate or triplicate and are shown as picomoles per $1x10^6$ cells. Box and whisker plots indicate median, $25^{th}$ and $75^{th}$ percentile and range of data. Each sample is indicated with a different symbol, all data points are shown. Significance determined by one-way ANOVA (Tukey's multiple correction). C-I sample was not included in statistical analysis. Significance values for 2-hydroxyglutarate: $P$ = 0.0001 (IDH1 vs WT) and 0.0006 (IDH2 vs WT); fumarate: $P$ = 0.0321 (IDH1 vs WT); malate: $P$ = 0.0083 (IDH1 vs WT) and 0.0091 (IDH2 vs WT). Details of patient samples are provided in Supplementary Data 5. Source data are provided in Source Data file.

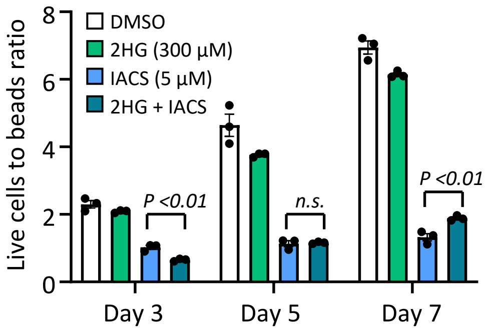


**Supplementary Figure 4. Combination treatment of THP1 cells.** THP1 cells cultured over 7 days in the presence of DMSO, 300 µM exogenous ocytl-(R)-2HG, 5 µM IACS-010759 (IACS) or the combination. Data shown as the mean +/- SEM of 3 independent experiments. Each dot represents an independent experiment. Significance between IACS alone and IACS in combination with 2HG was determined by unpaired two-tailed t-test. Significance values for day 3: *P* = 0.0045, day 5: not significant (n.s.), day 7: *P* = 0.0083. Source data are provided in Source Data file.

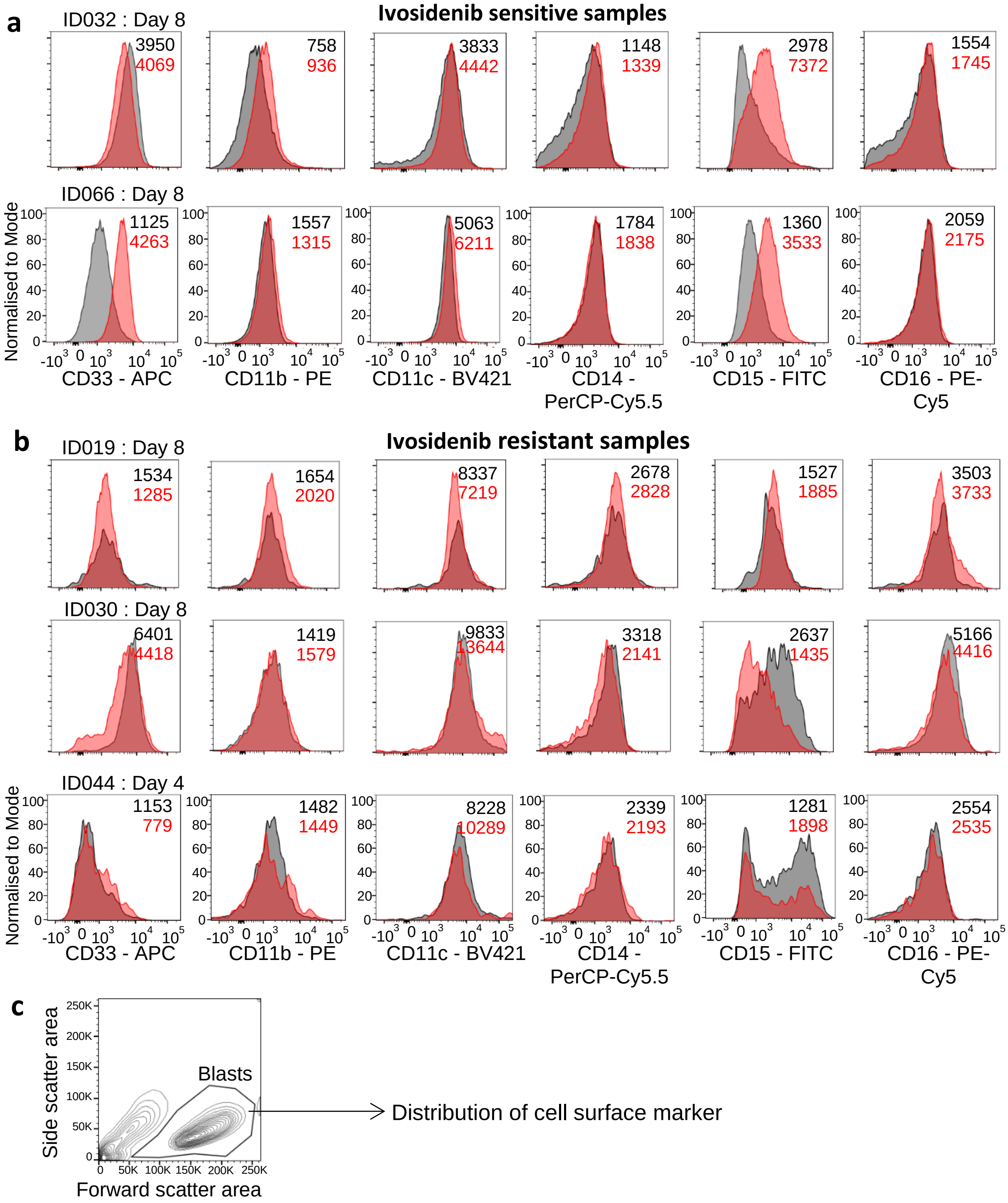


**Supplementary Figure 5. Primary response of *IDH1*-mutant AML samples to ivosidenib.** **(a)** Sensitivity and **(b)** resistance to ivosidenib determined by expression of lineage specific cell surface markers in response to 10 µM ivosidenib treatment (red) relative to DMSO (grey) for a panel of IDH1 mutant AML samples. Data analysed with FloJo™ and shown normalised to mode. Mean fluorescence intensity (MFI) is shown for both DMSO (in black) and ivosidenib (in red). Details of patient samples are provided in Supplementary Data 5. **(c)** Schematic of gating strategy for analysis. The blast cell population was gated on forward scatter area versus side scatter area and mean fluorescence intensity (MFI) of the cell population for each cell surface marker was recorded. Graphs of fluorescence distribution were presented normalised to mode for visualisation.

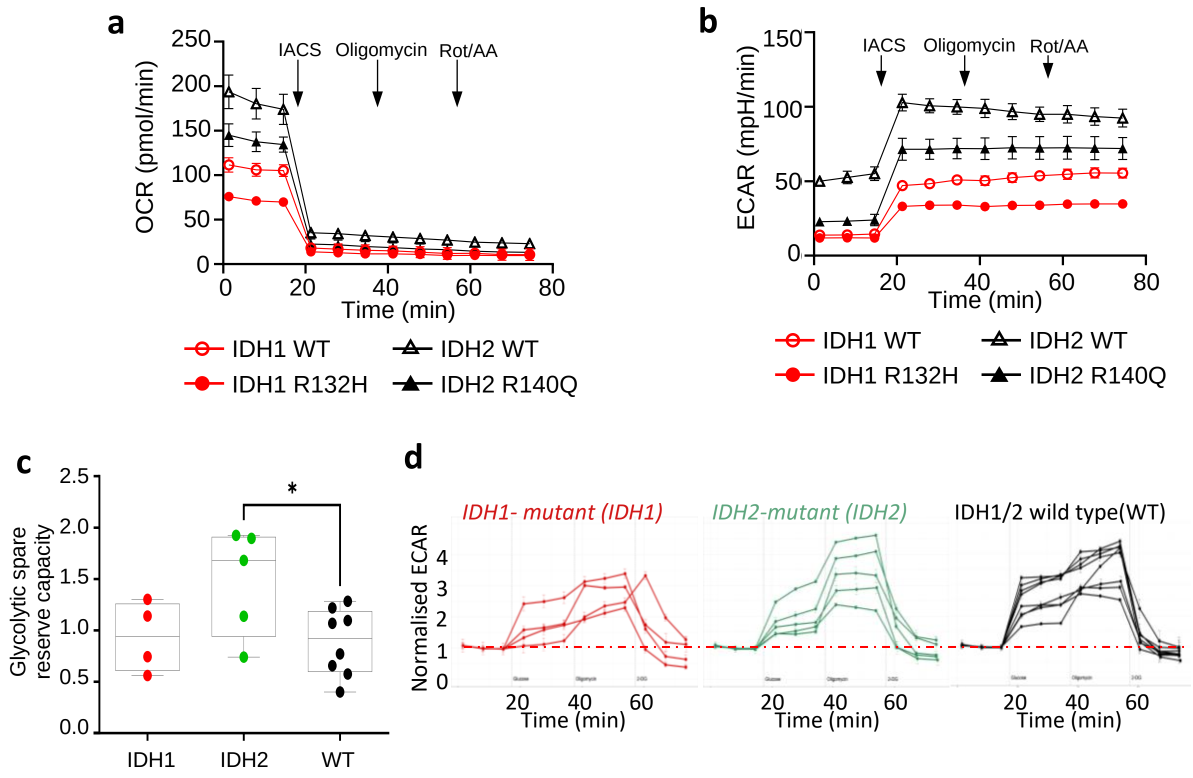


**Supplementary Figure 6. Glycolysis capacity in isogenic THP1 cells and primary AML samples. (a)** Raw oxygen consumption rate (OCR) and **(b)** extracellular acidification rate (ECAR) per $5x10^4$ of THP1 cells expressing IDH1 R132H, IDH2 R140Q or respective wild type (WT) controls with addition of 5 µM IACS-010759 at first injection. Each data point represents the mean +/- SD of 6 replicate reads for each sample. Data relates to Figure 4a-c. IACS, IACS-010759; Rot, rotenone; AA, antimycin A. **(c)** Glycolytic spare reserve capacity (derived from normalized ECAR) measured in *IDH1*- (n=4), *IDH2*-mutant AML (n=5) or *IDH1/2* wild type (WT, n=8, also wild type for complex-I, *FLT3*, *DNMT3A* and *NPM1*) samples. Significance determined by one-way ANOVA (Dunnet's post-hoc), * $P = 0.0368$. Box and whisker plots indicate median, $25^{th}$ and $75^{th}$ percentile and range of data. Each dot indicates a different patient sample. **(d)** Normalised ECAR for the individual samples summarised in (c). Each line represents an independent patient sample tested in triplicate and each value is the mean of a given measurement timepoint. Error bars show +/- SD. Data represented as fold change relative to basal ECAR. Details of patient samples used in c-d are provided in Supplementary Data 5. Source data are provided in Source Data file.

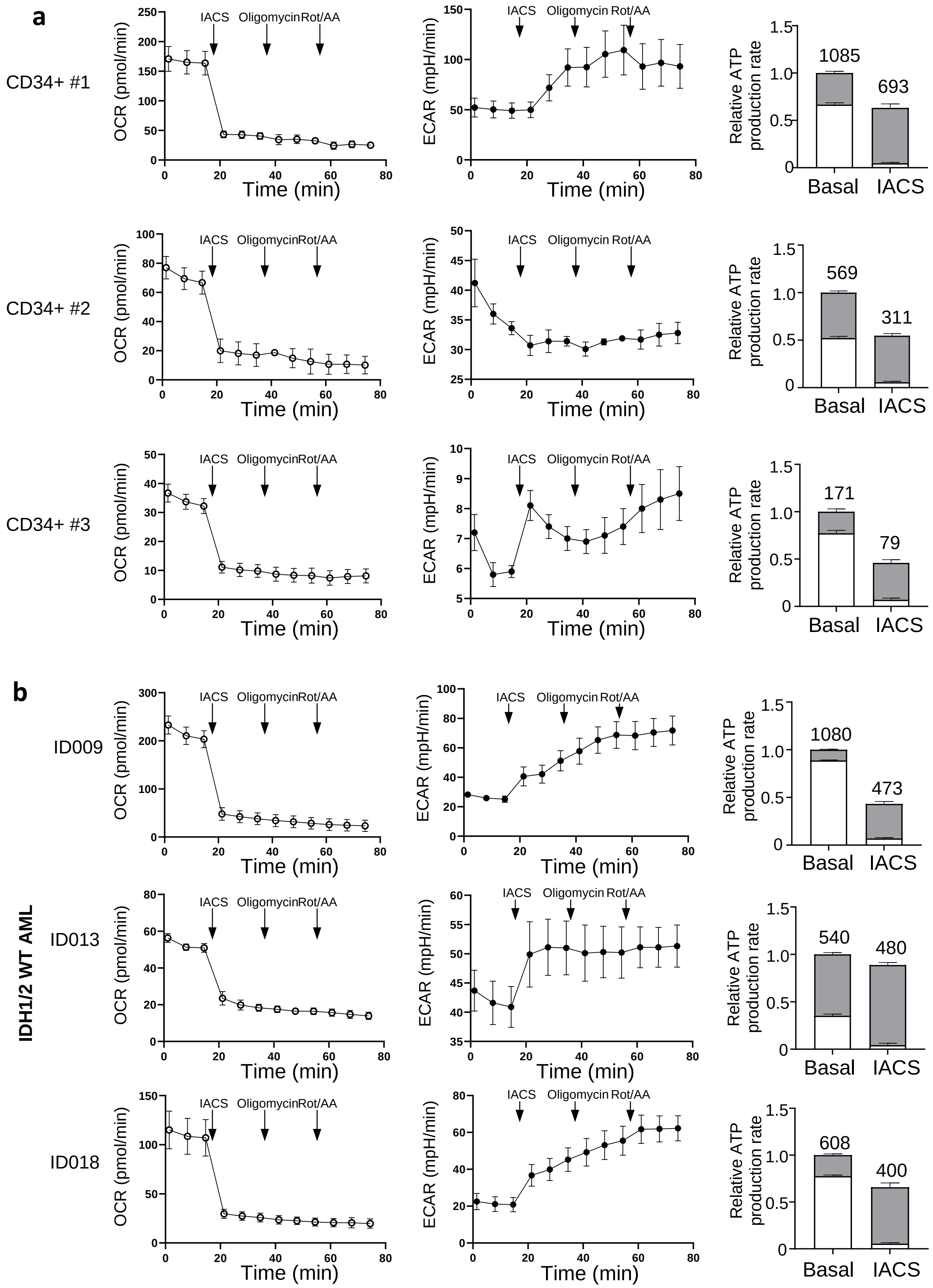
a
CD34+ #1
CD34+ #2
CD34+ #3
b
IDH1/2 WT AML
ID009
ID013
ID018
IACS
Oligomycin
Rot/AA
OCR (pmol/min)
ECAR (mpH/min)
Time (min)
Relative ATP production rate
Basal
IACS
1085
693
569
311
171
79
1080
473
540
480
608
400

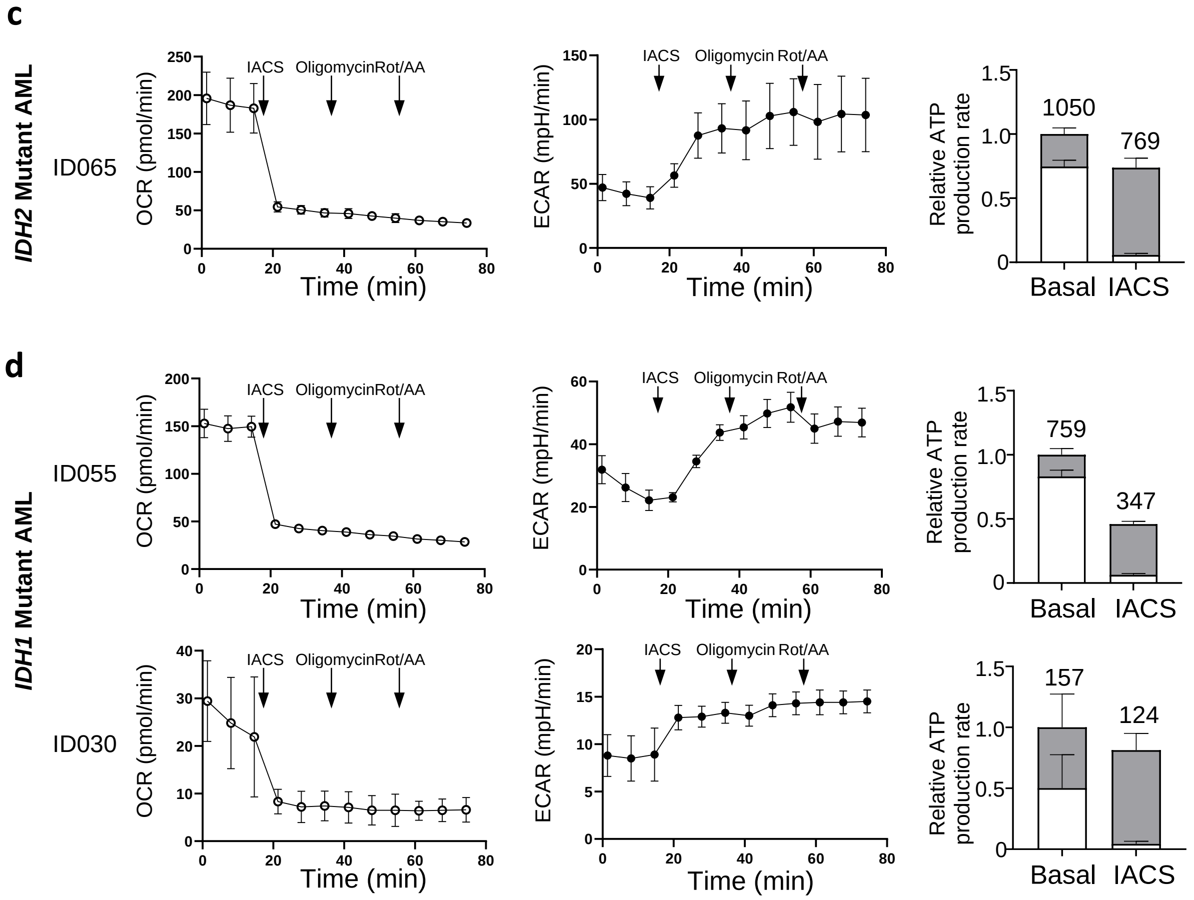


**Supplementary Figure 7. Glycolysis capacity and metabolic plasticity in primary AML samples.** Individual sample raw data related to Figure 4d. Raw oxygen consumption rate (OCR, left panel), extracellular acidification rate (ECAR, middle panel) and ATP production rate (right panel) following addition of 5 µM IACS-010759 (IACS), determined by ATP rate assay for **(a)** healthy CD34+ samples (n=3), **(b)** *IDH1/2* WT AML (n=3), **(c)** *IDH2*-mutant AML (n=1), and **(d)** *IDH1*-mutant AML(n=2). For OCR and ECAR plots, each data point represents the mean +/- SD of at least 4 technical replicate reads. ATP production rate is shown relative to basal total ATP production rate and values above bars are total ATP production rates (pmol/min) under each condition. Contribution to ATP production rate from glycolysis (grey) and OXPHOS (white) are shown as mean, and error bar show +/- SD of at least 4 technical replicates. For clarity, only the upper half of the error bar is shown. Details of patient samples are provided in Supplementary Data 5. Source data are provided in Source Data file.

**Supplementary References**


1 Cancer Genome Atlas Research, N. *et al.* Genomic and epigenomic landscapes of adult de novo acute myeloid leukemia. *The New England journal of medicine* **368**, 2059-2074, doi:10.1056/NEJMoa1301689 (2013).

2 Yang, J. *et al.* The I-TASSER Suite: protein structure and function prediction. *Nat Methods* **12**, 7-8, doi:10.1038/nmeth.3213 (2015).
