# Supplementary material for: Germline mutations in mitochondrial complex I reveal genetic and targetable vulnerability in IDH1-mutant acute myeloid leukaemia
Source: Nat Commun. 2022 May 12;13:2614. doi: 10.1038/s41467-022-30223-9 (PMC9098909; doi:10.1038/s41467-022-30223-9)
Supplement: Supplementary file 3 — Description of Additional Supplementary Files [file 41467_2022_30223_MOESM3_ESM.pdf]

Description of Additional Supplementary Files

File name: Supplementary Data 1
Description: Variants identified (MAF <0.005) in whole-exome sequencing screen of diagnostic AML samples.

File name: Supplementary Data 2
Description: Cohort characteristics associated with mitochondrial respiratory chain variants (whole exome sequencing cohort).

File name: Supplementary Data 3
Description: Variants identified (MAF <0.005) in the ion-torrent sequencing screen of diagnostic AML samples.

File name: Supplementary Data 4
Description: Cohort characteristics table for complex-I mutated AML (whole-exome sequencing and ion-torrent cohorts).

File name: Supplementary Data 5
Description: Primary AML samples used in functional assays.

File name: Supplementary Data 6
Description: Primer sequences used in Ion Torrent sequencing.

File name: Supplementary Movie 1
Description: Structural modelling of NDUFS8 p.Arg2 within complex-I of the mitochondrial respiratory chain. Related to Supplementary Figure 2e-f
