# Supplementary material for: Germline mutations in mitochondrial complex I reveal genetic and targetable vulnerability in IDH1-mutant acute myeloid leukaemia
Source: Nat Commun. 2022 May 12;13:2614. doi: 10.1038/s41467-022-30223-9 (PMC9098909; doi:10.1038/s41467-022-30223-9)
Supplement: Supplementary file 11 — Reporting summary [file 41467_2022_30223_MOESM11_ESM.pdf]

nature portfolio

Corresponding author(s): Richard D'Andrea

Last updated by author(s): Feb 10, 2022

Reporting Summary

Nature Portfolio wishes to improve the reproducibility of the work that we publish. This form provides structure for consistency and transparency in reporting. For further information on Nature Portfolio policies, see our Editorial Policies and the Editorial Policy Checklist.

Statistics

For all statistical analyses, confirm that the following items are present in the figure legend, table legend, main text, or Methods section.

n/a	Confirmed	
☐	☒	The exact sample size (n) for each experimental group/condition, given as a discrete number and unit of measurement
☐	☒	A statement on whether measurements were taken from distinct samples or whether the same sample was measured repeatedly
☐	☒	The statistical test(s) used AND whether they are one- or two-sided<br>*Only common tests should be described solely by name; describe more complex techniques in the Methods section.*
☒	☐	A description of all covariates tested
☐	☒	A description of any assumptions or corrections, such as tests of normality and adjustment for multiple comparisons
☐	☒	A full description of the statistical parameters including central tendency (e.g. means) or other basic estimates (e.g. regression coefficient) AND variation (e.g. standard deviation) or associated estimates of uncertainty (e.g. confidence intervals)
☐	☒	For null hypothesis testing, the test statistic (e.g. F, t, r) with confidence intervals, effect sizes, degrees of freedom and P value noted<br>*Give P values as exact values whenever suitable.*
☒	☐	For Bayesian analysis, information on the choice of priors and Markov chain Monte Carlo settings
☒	☐	For hierarchical and complex designs, identification of the appropriate level for tests and full reporting of outcomes
☒	☐	Estimates of effect sizes (e.g. Cohen's d, Pearson's r), indicating how they were calculated

Our web collection on statistics for biologists contains articles on many of the points above.

Software and code

Policy information about availability of computer code

Data collection	No software was used for data collection.
Data analysis	Data processing and statistical analysis was performed in R(v3.5) using base R function calls. The R package of Weighted exclusivity test was downloaded from Github (https://github.com/jhrcook/wext). Statistical analysis was also performed using Graphpad Prism (v9). Protein crystal structures were visualized using Pymol (v2.1.0). Gene set enrichment analysis was performed using GSEA (v4.1.0). Differential expression was performed using R (v3.6.3) and edgeR (v3.3).

For manuscripts utilizing custom algorithms or software that are central to the research but not yet described in published literature, software must be made available to editors and reviewers. We strongly encourage code deposition in a community repository (e.g. GitHub). See the Nature Portfolio guidelines for submitting code & software for further information.

Data

Policy information about availability of data

All manuscripts must include a data availability statement. This statement should provide the following information, where applicable:

- Accession codes, unique identifiers, or web links for publicly available datasets
- A description of any restrictions on data availability
- For clinical datasets or third party data, please ensure that the statement adheres to our policy

The relevant data supporting the key findings of this study are available within the article and its Supplementary Information and Data files or from the corresponding author upon reasonable request. Source data are provided with this paper. The variant data generated in this study have been deposited in the EGA European Genome-Phenome Archive database with restricted access (due to HREC requirements). Application for access can be done directly through the EGA website (www.ega-archive.org). Additional information can be requested from the corresponding author.

Field-specific reporting

Please select the one below that is the best fit for your research. If you are not sure, read the appropriate sections before making your selection.

☒ Life sciences ☐ Behavioural & social sciences ☐ Ecological, evolutionary & environmental sciences

For a reference copy of the document with all sections, see nature.com/documents/nr-reporting-summary-flat.pdf

Life sciences study design

All studies must disclose on these points even when the disclosure is negative.

Sample size	Sample-size calculation was not performed. Sample size for data from AML primary samples was determined based on availability of clinical and molecular annotation, and availability of specimens.
Data exclusions	No data was excluded.
Replication	Biochemical and in vitro experiments were repeated 2/3 times depending on availability of cellular material. Where data cannot be combined from independent experiments, representative experiments are shown. Characterisation of primary sample material are single experiments due to sample availability. Samples from multiple patients per group were tested, when adequate specimen was available. Mutual exclusivity of IDH1-mutation and complex-I mutations are presented for 3 cohorts.
Randomization	Allocation is not random. Allocation of samples to experimental groups are defined by clinical and molecular characteristics.
Blinding	Blinding is not applicable as no results required subjective investigator scoring.

Reporting for specific materials, systems and methods

We require information from authors about some types of materials, experimental systems and methods used in many studies. Here, indicate whether each material, system or method listed is relevant to your study. If you are not sure if a list item applies to your research, read the appropriate section before selecting a response.

Materials & experimental systems

n/a	Involved in the study
☐	☒ Antibodies
☐	☒ Eukaryotic cell lines
☒	☐ Palaeontology and archaeology
☒	☐ Animals and other organisms
☐	☒ Human research participants
☒	☐ Clinical data
☒	☐ Dual use research of concern

Methods

n/a	Involved in the study
☒	☐ ChIP-seq
☐	☒ Flow cytometry
☒	☐ MRI-based neuroimaging

Antibodies

Antibodies used	For flow cytometry:<br>anti-human CD33-APC (P67-6) BD Biosciences, Cat # 340474, 1:25 dilution<br>anti-human CD11b-PE (D12) BD Biosciences, Cat # 347557, 1:25 dilution<br>anti-human CD11c-BV421 (B-ly6) BD Biosciences, Cat # 562561, 1:25 dilution<br>anti-human CD14-PerCP-Cy5.5 (M5E2) BD Biosciences, Cat # 550787, 1:25 dilution<br>anti-human CD15-FITC (MMA) BD Biosciences, Cat # 347423, 1:25 dilution<br>anti-human CD16-PE-Cy5 (3G8) BD Biosciences, Cat # 555408, 1:25 dilution<br><br>For western blot:<br>NDUFS8 (D-5) mouse monoclonal for western blot, Santa Cruz Biotechnology, Cat # sc-515537, 1:2000 dilution<br>b-Actin (ACTBD11B7) mouse monoclonal for western blot, Santa Cruz biotechnology, Cat # sc-81178, 1:1000 dilution
Validation	All of the antibodies used have been extensively utilized in the literature and have been validated previously.

Eukaryotic cell lines

Policy information about cell lines

Cell line source(s)	THP-1 cells and HEK293T cells were sourced from ATCC (TIB-202 and CRL-3216, respectively).

Authentication	THP-1 cell line was confirmed by short tandem repeat analysis. HEK293T cell line was not authenticated.
Mycoplasma contamination	THP-1 cells have been tested and are negative for mycoplasma contamination. HEK293T cells were not tested.
Commonly misidentified lines (See ICLAC register)	No commonly misidentified lines were used.

Human research participants

Policy information about studies involving human research participants

Population characteristics	Patient cohorts that were analysed have been described previously or have been described in supplementary data files. See references in-text.
Recruitment	No recruitment of participants specifically for this project. Biospecimens used in this study are collected for research with written, informed consent. AML patient samples were donated at the time of routine diagnostic and follow-up assessments and no compensation was paid. Volunteer healthy donors were paid an honorarium for their donation to research.
Ethics oversight	Royal Adelaide Hospital and University of South Australia (Adelaide), Princess Alexandra/Metro South Health University of Queensland (Brisbane), Stanford University (California).

Note that full information on the approval of the study protocol must also be provided in the manuscript.

Flow Cytometry

Plots

Confirm that:

- [x] The axis labels state the marker and fluorochrome used (e.g. CD4-FITC).
- [x] The axis scales are clearly visible. Include numbers along axes only for bottom left plot of group (a 'group' is an analysis of identical markers).
- [x] All plots are contour plots with outliers or pseudocolor plots.
- [x] A numerical value for number of cells or percentage (with statistics) is provided.

Methodology

Sample preparation	Lymphoid-depleted patient AML samples were cultured in IMDM supplemented with 20% fetal calf serum, 50 ng/ml human stem cell factor and 10 ng/ml each of thrombopoeitin, FLT-3 ligand, interleukin-3, interleukin-6 and granulocyte-colony stimulatory factor (Peprotech), 100 µM β-mercaptoethanol (BME, Sigma Aldrich) together with 10 µM ivosidenib or DMSO (control) for up to 8 days. Expression of lineage markers were determined on viable cells.
Instrument	BD FACSCanto™II
Software	FloJo™
Cell population abundance	Routinely FACS sorting was not performed to purify AML blasts. Lymphocyte and dead cell depletion was performed by magnetic bead separations (Miltenyi Biotec). The percentage of viable blasts >95% was confirmed by flow cytometry prior to cell differentiation assays, Seahorse assays, NADPH quantitation and IACS experiments.
Gating strategy	For viability, blast gate was determined from standard CD45-side scatter profile from healthy normals, as per a routine diagnostic pathology laboratory workflow. The viable blast gate was determined as the mid-point between the DAPI positive and DAPI negative populations. For differentiation, positivity for all myeloid maturation markers was determined by unstained and isotype controls set at 1% in the negative gate. Myeloid maturation markers were determined in the blast population determined by forward and side scatter distribution - this is shown in the relevant supplementary figure.

- [x] Tick this box to confirm that a figure exemplifying the gating strategy is provided in the Supplementary Information.
